# Supplementary material for: Patterns of Intron Gain and Loss in Fungi
Source: PLoS Biol. 2004 Nov 30;2(12):e422. doi: 10.1371/journal.pbio.0020422 (PMC532390; doi:10.1371/journal.pbio.0020422)
Supplement: Table S1 — Also available at http://genes.mit.edu/NielsenEtAl/. (4.3 MB ZIP). [file pbio.0020422.st001.zip › NielsenEtAl/html/974.html]

AN6542.1.NCU04173.1.MG03982.2.FG07335.1


```
 CLUSTAL W (1.82) Multiple Sequence Alignments - Introns Inserted


Sequence 1: AN6542.1	376 aa
Sequence 2: NCU04173.1	381 aa
Sequence 3: FG07335.1	380 aa
Sequence 4: MG03982.2	356 aa
Alignment Length: 381 aa
Number Identitical Residues: 341 aa
Alignment Score (without introns) 13160


MG03982.2 	------MEE1EVAALVIDNG2SGMCKAGFAGDDAPRAVFP1SIVGRPRHHG2IMIGMGQK
NCU04173.1	MIHAFPPTE~EVAALVIDNG2SGMCKAGFAGDDAPRAVFP1SIVGRPRHHG2IMIGMGQK
FG07335.1 	-MTYLCFTE~EVAALVIDNG2SGMCKAGFAGDDAPRAVFP1SIVGRPRHHG2IMIGMGQK
AN6542.1  	-----MPTE~EVAALVIDNG2SGMCKAGFAGDDAPRAVFP1SIVGRPRHHG2IMIGMGQK
          	        * ********** ******************* ********** ********

MG03982.2 	DSYVGDEAQSKRGILTLRYPIEHGVVTNWDDMEKIWHHTFYNELRVAPEEHPVLLTEAPI
NCU04173.1	DSYVGDEAQSKRGILTLRYPIEHGVVTNWDDMEKIWHHTFYNELRVAPEEHPVLLTEAPI
FG07335.1 	DSYVGDEAQSKRGILTLRYPIEHGVVTNWDDMEKIWHHTFYNELRVAPEEHPVLLTEAPI
AN6542.1  	DSYVGDEAQSKRGILTLRYPIEHGVVTNWDDMEKIWHHTFYNELRVAPEEHPVLLTEAPI
          	************************************************************

MG03982.2 	NPKSNREKMTQIVFETFNAPAFYVSIQAVLSLYASGRTTGIVLDSGDGVTHVVPIYEGFS
NCU04173.1	NPKSNREKMTQIVFETFNAPAFYVSIQAVLSLYASGRTTGIVLDSGDGVTHVVPIYEGFA
FG07335.1 	NPKSNREKMTQIVFETFNAPAFYVSIQAVLSLYASGRTTGIVLDSGDGVTHVVPIYEGFA
AN6542.1  	NPKSNREKMTQIVFETFNAPAFYVSIQAVLSLYASGRTTGIVLDSGDGVTHVVPIYEGFA
          	***********************************************************:

MG03982.2 	LPHAIARVDMAGRDLTDYLMKILAERGYTFSTTAEREIVRDIKEKLCYVALDFEQEIQTA
NCU04173.1	LPHAIARVDMAGRDLTDYLMKILAERGYTFSTTAEREIVRDIKEKLCYVALDFEQEIQTA
FG07335.1 	LPHAIARVDMAGRDLTDYLMKILAERGYTFSTTAEREIVRDIKEKLCYVALDFEQEIQTA
AN6542.1  	LPHAISRVDMAGRDLTDYLMKILAERGYTFSTTAEREIVRDIKEKLCYVALDFEQEIQTA
          	*****:******************************************************

MG03982.2 	AQSSSLEKSYELPDGQ0SWVLR--------AVVS-----------HVTTFNSIMKCDVDV
NCU04173.1	AQSSSLEKSYELPDGQ~VITIGNERFRAPEALFQPSVLGLESGGIHVTTFNSIMKCDVDV
FG07335.1 	AQSSSLEKSYELPDGQ~VITIGNERFRAPEALFQPSVLGLESGGIHVTTFNSIMKCDVDV
AN6542.1  	SQSSSLEKSYELPDGQ~VITIGNERFRAPEALFQPSVLGLESGGIHVTTFNSIMKCDVDV
          	:***************   .: ..   :..*:...:  . .:.. ***************

MG03982.2 	RKDLYNNIVM0SGGTTMYPGLSDRMQKEITALAPSSMKVKIIAPPERKYSVWIGGSILAS
NCU04173.1	RKDLYGNIVM0SGGTTMYPGLSDRMQKEITALAPSSMKVKIIAPPERKYSVWIGGSILAS
FG07335.1 	RKDLYGNIVM0SGGTTMYPGLSDRMQKEITALAPSSMKVKIIAPPERKYSVWIGGSILAS
AN6542.1  	RKDLYGNIVM0SGGTTMYPGISDRMQKEITALAPSSMKVKIIAPPERKYSVWIGGSILAS
          	*****.**** *********:***************************************

MG03982.2 	LSTFQQMWISKQEYDESGPSIVHRKCF
NCU04173.1	LSTFQQMWISKQEYDESGPSIVHRKCF
FG07335.1 	LSTFQQMWISKQEYDESGPSIVHRKCF
AN6542.1  	LSTFQQMWISKQEYDESGPSIVHRKCF
          	***************************
```
